# The Saudi-Arabic adaptation of the Body Shape Questionnaire (BSQ34): Psychometrics and norms of the full version and the short version (BSQ8C)

**DOI:** 10.3389/fpsyg.2022.1046075

**Published:** 2022-12-01

**Authors:** Bernou Melisse, Eric F. van Furth, Edwin de Beurs

**Affiliations:** ^1^Novarum Center for Eating Disorders and Obesity, Amstelveen, Netherlands; ^2^Section Clinical Psychology, Leiden University, Leiden, Netherlands; ^3^GGZ Rivierduinen, Leiden, Netherlands; ^4^Department of Psychiatry, Leiden University Medical Center, Leiden, Netherlands; ^5^Research Department, Arkin Mental Health Institute, Amsterdam, Netherlands

**Keywords:** body shape questionnaire, psychometric properties, normative data, Saudi Arabia, body-shape dissatisfaction

## Abstract

**Introduction:**

Saudi Arabia experiences elevated levels of body-shape dissatisfaction which might be related to the increased thin ideal. Studies on body-shape dissatisfaction are scarce, mainly because adapted assessment tools are unavailable. This study describes the Saudi-Arabic adaptation of the Body Shape Questionnaire (BSQ34), preliminary examines the psychometric properties and provides normative data.

**Methods:**

The BSQ34 was administered in a convenience community sample (*N* = 867) between April 2017 and May 2018. Receiver-operating-characteristic curve analysis was used to establish discriminant validity, in a subsample (*N* = 602) in which the Eating Disorder Examination-Shape concern, was administered, the factor structure investigated with confirmatory-factor analyses and *T*-scores and percentile scores were determined.

**Results:**

The BSQ34 discriminated well between low and high levels of body-shape dissatisfaction (area-under-the-curve value = 0.93), had high internal consistency and a unidimensional factor structure, and 23.9% appeared at risk for body-shape dissatisfaction. Analyses were repeated for the shortened BSQ8C, which yielded similar results.

**Discussion:**

The results indicated that the BSQ34 and BSQ8C appeared suitable measurement tools to screen for body-shape dissatisfaction in a Saudi convenience community sample, mainly comprised young, unmarried, and highly educated women. The BSQ34 supplies more information on the type of concerns respondents have, which is worthwhile when the measure is used in a clinical setting; the BSQ8C is recommended as a short screener. As body-shape dissatisfaction is viewed as a risk factor for the development of eating disorder symptoms, screening for body-shape dissatisfaction with reliable tools is important to detect individuals at risk for eating disorder symptoms and may suggest subsequent preventive steps.

## Introduction

Body-shape dissatisfaction, defined as “a subjective negative evaluation of one’s physical body,” is a maintaining factor of eating disorders (Stice, 2002). Recent studies report high prevalences of body-shape dissatisfaction in parts of the Arabic world, but the current prevalence of body-shape dissatisfaction in Saudi Arabia remains unknown, as only one study reported that 83% of Saudi women preferred a different weight (Rasheed, 1998). However, approximately 30–78% of women and 30–58% of men in the Gulf are reported to be dissatisfied with their bodies (Al-Sendi et al., 2004; Bener et al., 2006; Eapen et al., 2006; Thomas et al., 2010). Saudi Arabia currently experiences major sociocultural changes, adopting Western values (Thomas et al., 2018), and the thin ideal increased in Saudi Arabia (Thomas et al., 2010), potentially explaining the elevated levels of body-shape dissatisfaction.

The increasing prevalence of body-shape dissatisfaction might not only be due to the shift in beauty ideals from a curvy body into a thin body (Melisse et al., 2020a), but also the presence of single-sex schools in Saudi Arabia could influence body perception (Dittmar, 2005). The social comparison theory states that individuals gather information about themselves and where they fit in society by comparing themselves to more attractive peers, which is associated with body-shape dissatisfaction (Dittmar, 2005; Carter, 2022). In addition, self-concept in women is mainly determined by appearance and its evaluation by others (Dittmar, 2005). Furthermore, high levels of exposure to idealized body images, enhanced by single-sex schools, often accumulate into body-shape dissatisfaction (Frederick et al., 2017; ALAhmari et al., 2019). Moreover, the number of people with excess weight is on the rise in Saudi Arabia, which might also result in an increase of body-shape dissatisfaction (Melisse et al., 2020b). Even though body-shape dissatisfaction is prevalent, its consequences are often underestimated. For instance, Saudis who are dissatisfied with their bodies have an increased risk for unhealthy dietary habits (Stice and Shaw, 2002), which may result in developing eating disorder symptoms, as a strong association was found between body-shape dissatisfaction and eating disorder symptoms in Saudi Arabia (Melisse et al., 2022). Furthermore, eating disorder symptoms are associated with psychological symptoms in Saudi Arabia (AlHadi et al., 2022), and body-shape dissatisfaction is associated with psychological symptoms in other cultures (Rodríguez-Cano et al., 2006; Rich and LeClere, 2011; Murray et al., 2013; Gailledrat et al., 2016; Pritchard et al., 2021; Turk et al., 2021).

Research on body-shape dissatisfaction in Saudi Arabia is hampered, by the unavailability of assessment tools. Thus, adapted assessment tools for the measurement of body-shape dissatisfaction are urgently needed for the Saudi population. The Body Shape Questionnaire (BSQ; 34 items; BSQ34; Cooper et al., 1987) is most often used to measure body-shape dissatisfaction (Rosen et al., 1996). In addition, various short versions of the BSQ34 (16A, 16B, 14, 8A, 8B, 8C, 8D) are evaluated (Kapstad, 2015), of which the 8C version appears superior over other short versions, as it shows high sensitivity to change during therapy (Pook, 2008). Both, the BSQ34 and BSQ8C are adapted for use in various western, Latin (Rosen et al., 1996; Pook, 2008; Welch et al., 2012; da Silva et al., 2014; Kapstad, 2015), and Iraqi-Arab cultures (Medya and Ishak, 2016) and have strong psychometric properties, such as high internal validity, test–retest reliability, convergent validity, and unidimensional factor structure. Furthermore, the BSQ34 and BSQ8C are currently not available in an Arabic version adapted for use in Saudi Arabia.

The ability to screen for body-shape dissatisfaction is an important first step to help prevent development of eating disorder symptoms and other psychological symptoms through intervention programs, since targeted programs are more effective than universal preventative programs (Stice et al., 2019). In order to select participants for such preventative programs the BSQ8C can be used as a first screener before administration of the BSQ34 among those who scored above cut-off on the BSQ8C. In addition, a valid Saudi- Arabic BSQ could be used to measure reduction of eating disorder symptoms after eating disorder treatment. Furthermore, based on an Item Response Theory (IRT) analysis, factor scores can be used to obtain normalized standard scores (*T*-scores) and to establish percentile scores, both will offer a conversion of raw scores into these common metrics, which will ease interpretation and increase applicability of the measure (de Beurs et al., 2022a).

The aim of this brief report is to evaluate the psychometric properties (internal consistency, concurrent and discriminative validity, and factor structure) of a Saudi-Arabic version of the BSQ34, and the BSQ8C and investigate the screening potential for body-shape dissatisfaction in a convenience community sample in Saudi Arabia. The additional aim is to establish norms, *T*-scores, and percentile scores as they enable to measure whether body-shape dissatisfaction changes over time. The community sample first completes an online BSQ34, and when they leave their contact details they will be contacted to participate in a brief in-person interview.

## Materials and methods

### Procedure

In order to validate the Arabic BSQs adapted for use in Saudi Arabia, a convenience sample was recruited as Saudis are very sensitive to how they are viewed by others, and therefore, less likely to participate in surveys, questioning them on their personal beliefs and values (Al-Darmaki, 2003). Therefore, the aim was to reach as many Saudi passport holders as possible. Recruitment took place between April 2017 and May 2018 from students [Princess Noura University (PNU), King Saud University in Riyadh], and through social media (Twitter, Facebook), and the social network of the first author (BM; friends, colleagues, and their relatives and friends). Furthermore, some of BM’s students recruited participants through their personal network. Participants had to be Saudi, literate, and aged≥18. Participants provided informed consent and completed anonymously an online self-report questionnaire including the BSQ34 and demographics through Survey Monkey (Waclawski, 2012). At a second phase of data collection, between November 2017 and May 2018, the EDE-SC interview was administered to a subsample (*N* = 602). Participants who provided their contact details in the online BSQ34 were contacted for an EDE-SC interview.

### Participants

The study was approved on 7 May 2017 (17–0097) by the ethical board of PNU. A total of 871 Saudis were recruited of which four participants (0.4%) had ≥5% missing data regarding BSQ34 items and were therefore excluded, resulting in a sample size of *N* = 867. The mean age was 23.6 (*SD* = 5.5) years, and the majority (*n* = 475, 54.8%) was aged between 18 and 21 years old. There were several differences in the study sample compared to the general Saudi population: the majority were women (85.5 vs. 42.3%), university student (41.2 vs. 4.4%), and unmarried (76.5 vs. 33.0%). Body Mass Index was calculated based on self-reported body weight and height (*M* = 25.08, *SD* = 6.8). Table 1 displays demographics of the sample.

**Table 1:** Demographics of a Saudi convenience sample (*N* = 867).

		*N*	*M (SD)*
Age		867	23.6 (5.5)
	18–21 years	475 (54.8%)	
	22–25 years	162 (18.7%)	
	26–40 years	175 (20.2%)	
	41–81 years	54 (6.2%)	
Gender			
	Female	745 (85.5%)	
	Male	122 (14.5%)	
			
BMI		867	25.2 (6.8)
Marital status		867	
	Married	149 (17.2%)	
	Unmarried	663 (76.5%)	
	Divorced	53 (6.1%)	
Occupation/ education		867	
	High school	232 (26.9%)	
	University in country of heritage	336 (38.7%)	
	University in Arab country	17 (2.0%)	
	University in Western country	43 (0.5%)	
	Employed	130 (14.9%)	
	Unemployed	90 (10.4%)	
	Other	57 (6.6%)	
Measures			
	EDE-SC	602	2.8 (1.6)
	BSQ34	867	86.5 (36.3)
	BSQ8C	867	21.3 (9.7)

BMI, body mass index; BSQ, body shape questionnaire; EDE-SC, eating disorder examination-shape concern subscale.

### Measures

The BSQ34 was administered and then compared to the Eating Disorder Examination-Shape Concern (EDE-SC) subscale to examine if the BSQ accurately measures body-shape dissatisfaction, as some studies state the BSQ measures shape concern (Cooper et al., 1987; da Silva et al., 2014) and some state it measures body-shape dissatisfaction (Welch et al., 2012; Kapstad, 2015) it is assumed that the EDE-SC and BSQ measure the same construct.

### Body shape questionnaire

The BSQ34 is a self-report questionnaire to measure body-shape dissatisfaction, such as fear of gaining weight, desire to lose weight, and self-devaluation related to physical-appearance, as experienced during the last 28 days. A total of 34 items are answered on a 6-point Likert scale (1, never, to 6:always; Cooper et al., 1987). The total score is the sum-score of all items and ranges between 34 and 204. The proposed cut-off score for the British original is <110, indicating body-shape dissatisfaction. A shortened ‘alternate’ form, comprising items 4, 6, 13, 16, 19, 23, 29, and 33, was proposed as the BSQ8C (Evans and Dolan, 1993) for which the cut-off score is <26 (Cooper et al., 1987). The BSQ34 and BSQ8C have good psychometric properties, such as high internal consistency (Cronbach’s *α* = 0.96, and 0.91, respectively), and good test–retest stability (*r* = 0.88, and 0.95, respectively; Rosen et al., 1996; Pook, 2008; Welch et al., 2012). A Jordan BSQ34 was provided by Mousa et al. (2010) which was slightly adapted by BM and a psychology student of PNU and a translator as Jordan and Saudi Arabic differ slightly. Differences were discussed and resolved. Then, a back translation was made by the translator. One cultural adaptation was made in question 27: as women in Saudi Arabia share cars rather than travel by bus, “bus seat” was changed to “car seat.”

A pilot study among 50 PNU Health faculty students conducted in January 2017 offered the choice of completing the English or Arabic version of the BSQ34, both versions were adapted for use in Saudi Arabia. Although bilingual, all students preferred the Arabic to the English BSQ34. Therefore, it was decided only to offer the Arabic version. Participant feedback on the pilot indicated that the quality of the translation was satisfactory.

### Eating disorder examination 16.0 shape concern scale

The shape concern scale of the Eating Disorder Examination (EDE-SC) consists of eight items measuring shape concern as a feature of eating disorders, is a subscale of a widely used semi-structured interview (EDE), which has good psychometric properties (Cooper et al., 1989). The EDE-SC assesses shape concern during the previous 28 days on a 7-point Likert scale (0: feature was absent, to 6: feature was markedly present/present every day; Cooper and Fairburn, 1987). Saudis with an EDE-SC score of <4.34 (community mean + 1SD) were considered high in shape concern, 117 participants (19.4%) scored within the clinical range. For the Arabic version adapted for use in Saudi Arabia, some items were first culturally adapted by BM and two of her students, then translated to Arabic, and back-translated by the students. In the item regarding discomfort about exposure, swimming and communal changing rooms were replaced by gym and weddings, and wearing a wider or dark-colored abaya (mandatory coat for women) was added since they were more appropriate for Saudi culture (Melisse et al., 2021). Internal consistency of the EDE-SC was high (Cronbach’s *α* = 0.87, McDonalds *ω* = 0.85), and an Exploratory Factor Analysis indicated a unidimensional factor structure for the shape concern subscale with item loadings between 0.50 and 0.87.

### Statistical analyses

The BSQ34 was compared to the EDE-SC subscale to examine if the BSQ34 accurately measures body-shape dissatisfaction. This procedure was repeated when analyzing only the eight items of the BSQ8C. A one-way ANOVA was conducted to test for the effect of gender, age, and occupation on BSQ score. Item scores were inspected regarding their mean (and SD) and the frequency distribution by assessing skewness and kurtosis. The unidimensionality of the BSQs was investigated with a Confirmatory-Factor Analysis (CFA). In addition, invariance of the BSQ34 and the BSQ8C across two age groups and genders (18–25 and 26–81) was investigated with a multi-group CFA measurement (Wu and Estabrook, 2016). Internal consistency of the BSQs was measured by Cronbach’s α (α ≥ 0.70 was considered good and α ≥ 0.90 excellent; Cronbach, 1951; Gliem and Gliem, 2003) as well as McDonald’s ω (MacDonald, 1999). In addition, an IRT-based transformation of scores was performed, as described elsewhere (de Beurs et al., 2022a,b). First, an IRT model was fitted to the data, and factor scores (theta’s) with *M* = 0 and SD = 1 were calculated. Next, these standard scores were converted into *T*-scores with *T* = 10*Z + 50. With curve fitting (Non-linear Least Squared; Baty et al., 2015), a function was derived to compute *T*-scores from raw scores. For *T*-scores a cut-off value of 55 was proposed. The appropriateness of this cut-off value for the BSQ34 and BSQ8C was investigated. The discriminative validity of the Saudi-BSQs was examined by a receiver-operating-characteristic (ROC) analysis. Thus, sensitivity and specificity of the BSQs were established regarding the presence of body-shape dissatisfaction/ shape concern as assessed by the EDE-SC. An EDE-SC score of 4.34 was used to distinguish between Saudis high and low in shape concern (Cooper and Fairburn, 1987). The area-under-the-curve (AUC) was calculated for both BSQs. An AUC ≥ 0.90 meant high accuracy, 0.70–0.90 moderate, and 0.50–0.70 low accuracy in predicting EDE-SC status. Data were analyzed with SPSS (version 28) and with *R* (package Lavaan, version 0.6–5) (Rosseel, 2012).

## Results

There was no effect of gender [*F*(1, 784) = 0.19*, p* = 0.659], occupation [*F*(6,732) = 1,46, *p* = 0.189], and no difference among four age groups up to 21, 22–25, 26–40, and 40–81 years old [*F*(3,785) = 1.60, *p* = 0.192]. Supplementary Table A shows that BSQ scores (BSQ34: *M* = 87.7, *SD* = 36.8; BSQ8C: *M* = 21.3, *SD* = 9.7) were slightly skewed due to an excess of low scores. Items 7, 8, 11, 13, 18, 19, 26, 27, 31, and 32 were skewed and peaked with many responses in the lowest response category (“never”). Internal consistency was high (BSQ34: Cronbach’s *α* = 0.96, McDonalds *ω* = 0.97; BSQ8C: Cronbach’s *α* = 0.85, McDonalds *ω* = 0.87). BSQ and EDE-SC scores were strongly correlated (BSQ34: *r* = 0.85, *p* < 0.001; BSQ8C: *r* = 0.82, *p* < 0.001). Table 2 shows that the ROC analysis revealed a high AUC (BSQ34: *AUC* = 0.93, 95%CI [0.90–0.95], *p* < 0.001; BSQ8C: *AUC* = 0.92, 95%CI [0.89–0.94], *p* < 0.001), which indicated that both Arabic BSQs adapted for use in Saudi Arabia discriminate well between individuals high and low in body-shape dissatisfaction according to the EDE-SC.

**Table 2:** Summary of reliability and validity measures of the Saudi BSQ34.

Measure	Mean	SD	*α*	*ω*	*R* (EDE-SC)	*AUC*	*n*, % above original cut-off^†^	*n*, % above estimated cut-off^‡^
BSQ34	87.7	36.8	0.96	0.97	0.85^*^	0.93	209, 23.9%	231, 26.7%
BSQ8C	21.3	9.7	0.85	0.87	0.82^*^	0.92	267, 30.8%	190, 21.9%

* *p* < 0.001.

† Original cut-off: BSQ34 < 110; BSQ8C < 26 (Cooper et al., 1987).

‡ Estimated cut-off: BSQ34 < 114; BSQ8C < 28.

*α* = Cronbach’s alpha (Cronbach, 1951); *ω* = Omega (hierarchical; MacDonald, 1999). AUC, area under the curve; BSQ, body shape questionnaire; EDE-SC, eating disorder. Examination- shape concern subscale

IRT analysis showed that a unidimensional model fitted well [*χ*^2^(527) = 2513.38; RMSEA = 0.069; 95%CI = 0.066–0.072; SRMSR = 0.063, TLI = 0.974, CFI = 0.976] for the BSQ34. Similar fit indexes were found for the BSQ8C. We also investigated with multi-group CFA whether the data yield sufficiently similar factor solutions among men and women and two age groups of respondents younger than 26 and 26 and older (Wu and Estabrook, 2016). We evaluated configural invariance, metric (weak factorial) invariance, and scalar (strong factorial) invariance. For both the BSQ34 and the BSQ8C, this provided models with almost equal fit, and no significant differences in fit were demonstrated. We concluded that the factorial structure is very similar for both age groups. Finally, we also investigated measurement invariance for gender and obtained similar results of equal fit for both genders. All in all, these results indicate that support for the unidimensional factor structures of the BSQ34 and BSQ8C is found in both age groups and for both genders.

**Figure 1:**
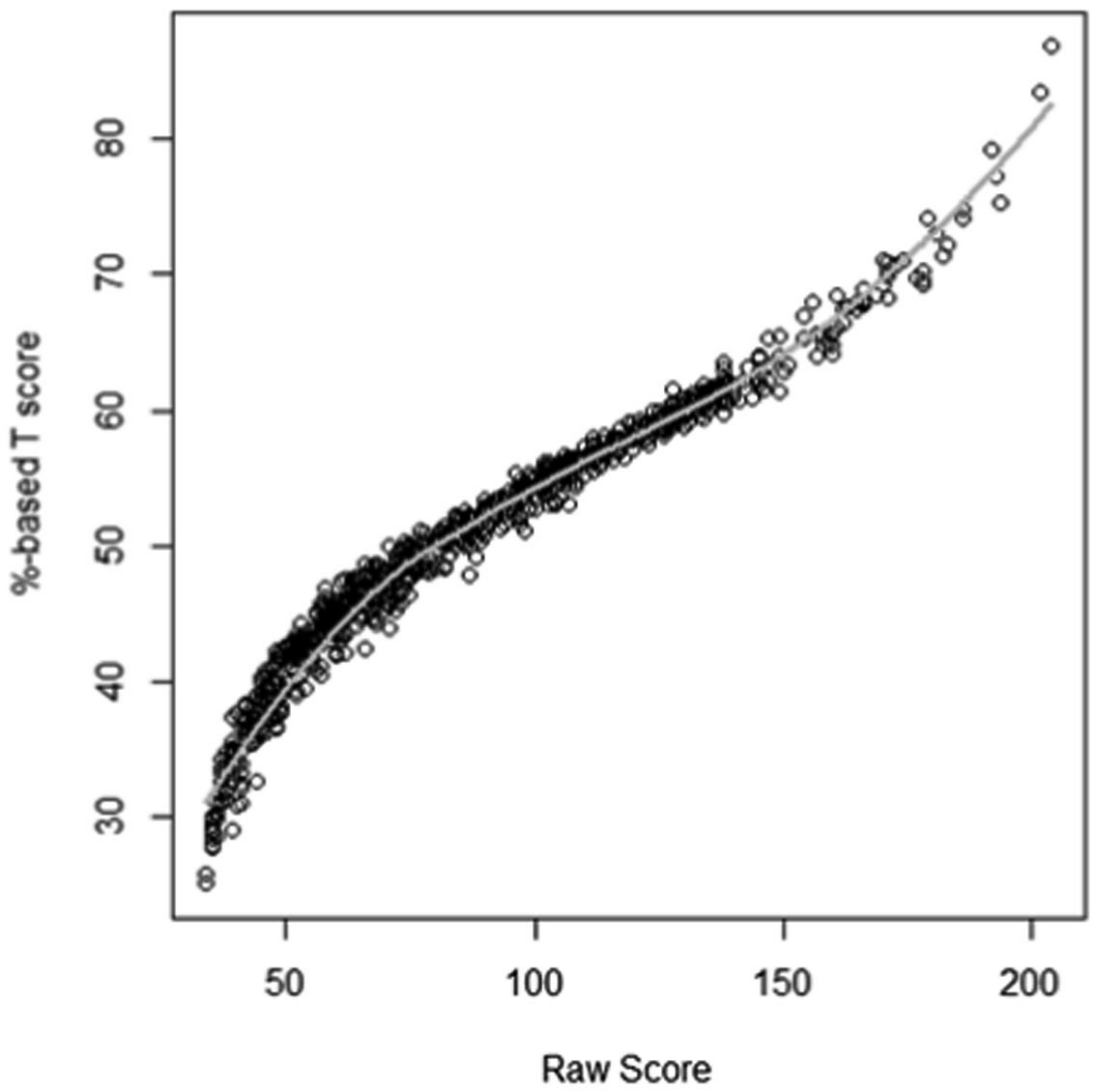
Relationship between raw scores and theta-based *T*-scores on the BSQ34.

**Figure 2:**
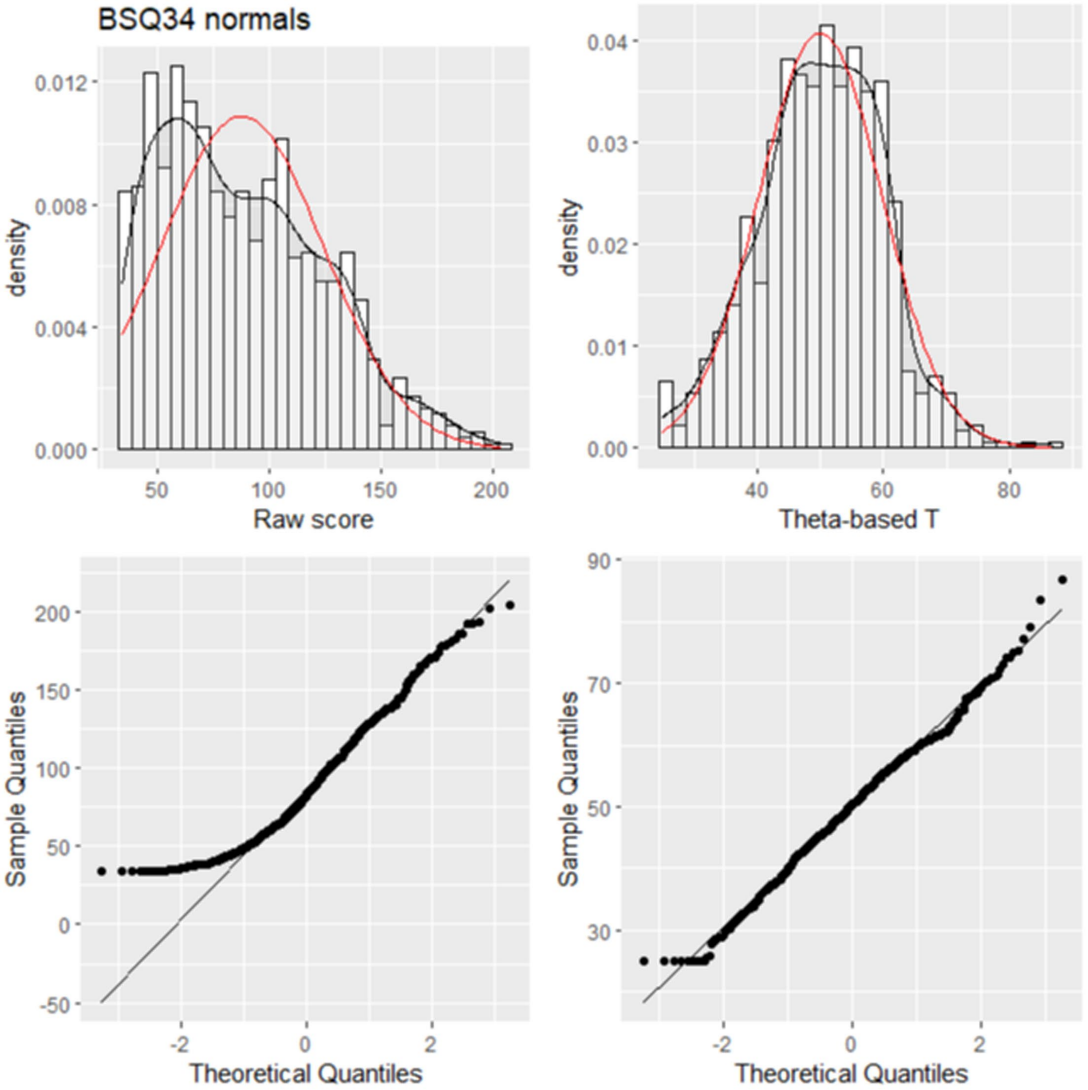
Histogram and normal probability plot for the BSQ34.

**Figure 3:**
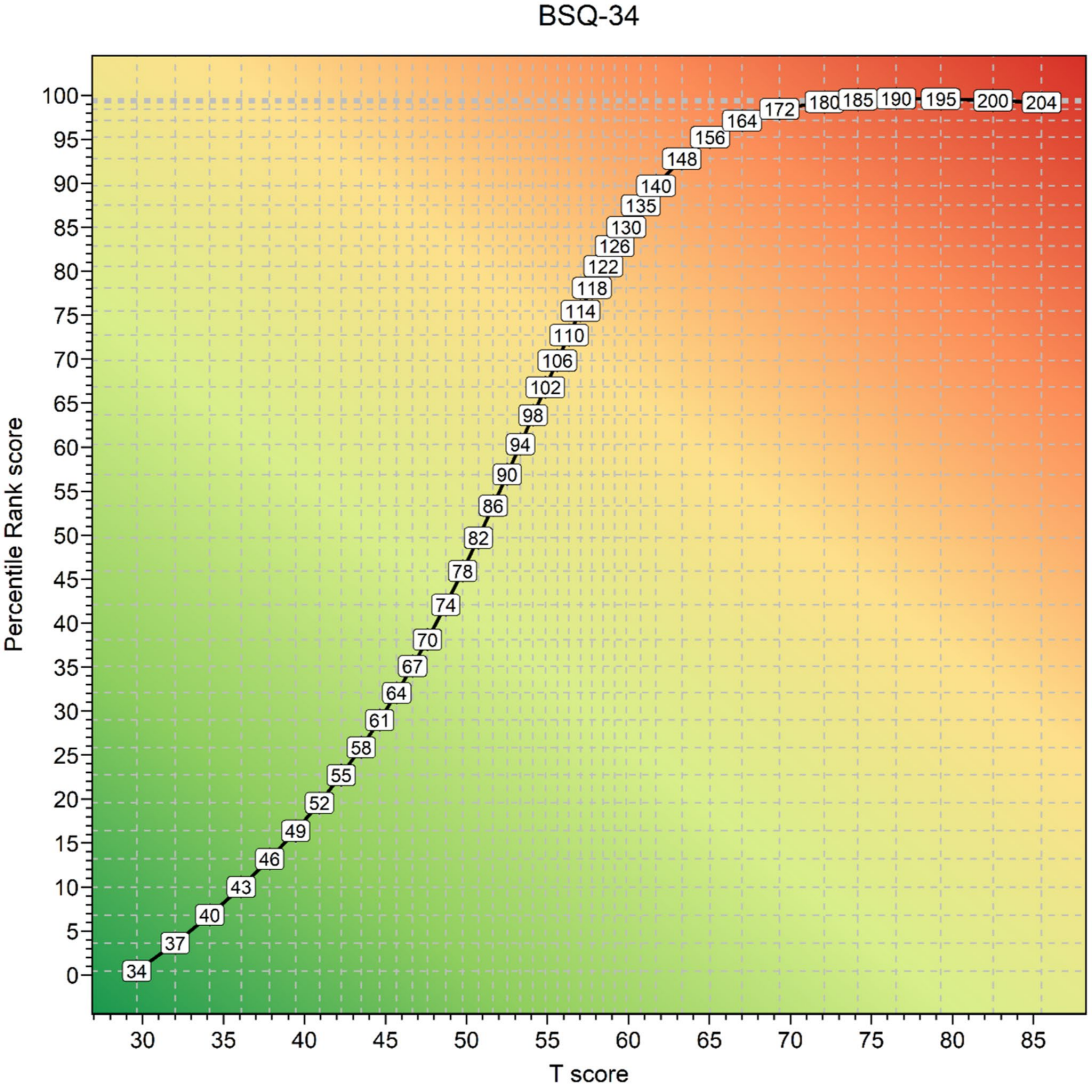
Raw scores, *T*-scores, and Percentile Rank scores of the Saudi-Arabic BSQ34.

Figure 1 shows the relation for the BSQ34 between raw scores and theta-based *T*-scores. The figure shows some variance in *T*-scores per raw score (vertical dispersion). Figure 2 shows histograms with a density line (black) and a normal curve (red) and normal probability plots for raw scores and *T*-scores of the BSQ34, showing that normalization was successful. We also established Percentile Rank (PR) scores based on the frequency of responses in the sample, using: $PR=\left(\frac{m+0.5k}{N}\right)*100$ , where m is the number of respondents with a score < Raw Score (RS), k is the number of respondents with exactly RS, and *N* is the size of the normative sample (Crawford and Garthwaite, 2009). Figure 3 shows for a selection of raw scores on the BSQ34, displayed on the curve, their association with *T*-scores and *PR* scores. In the supplementary materials cross-walk tables from RS to *T*-scores and percentile rank scores are provided (Supplementary Tables B, C). Finally, when the original cut-off score of <110 (Cooper et al., 1987) was applied to the BSQ34 sensitivity was 87.4%, and specificity 82.2%. However, the present data suggested different cut-off values. If optimal sensitivity is called for, e.g., when screening for subsequent assessment with a diagnostic interview, a raw score > 100 (*T* > 53.8) on the BSQ34 seems appropriate. When optimal specificity is called for, e.g., when screening for need of treatment, a cut-off of *RS* > 123 (*T* > 59.1) would seem more appropriate. Sensitivity and specificity are in balance at 0.85 when a cut-off of *RS* > 114 (*T* > 57.0) is applied. The BSQ8C cut-off values for RS can be found in Table 3; corresponding cut-offs in *T*-scores are *T* > 53.6, *T* > 57.7, and *T* > 56.4. Figure 2 shows a cross-walk figure to look up percentile rank scores and *T*-scores for a selection of raw scores.

**Table 3:** Sensitivity and specificity for the Saudi-Arabic body shape questionnaire.

Scale		Optimal sensitivity			Optimal specificity			Best of both			AUC	Cut-off	Sens.	Spec.	Cut-off	Sens.	Spec.	Cut-off	Sens.	Spec.
BSQ34	0.929	100	0.90	0.74	123	0.74	0.89	114	0.85	0.85
BSQ8C	0.916	24	0.89	0.72	30	0.74	0.88	28	0.83	0.83

AUC, area under the curve; Sens., sensitivity; Spec., specificity.

## Discussion

This is the first study to provide preliminary data on a measure to assess body-shape dissatisfaction in Saudi Arabia. The main findings of this study are that both versions of the Saudi- Arabic BSQ accurately discriminated between Saudis with low and high body-shape dissatisfaction, had a unidimensional factor structure, high internal consistency, and high convergent validity. Even though living conditions between men and women differed significantly (ALAhmari et al., 2019; Melisse et al., 2021), no effect of gender and level of education was found on BSQ34 or BSQ8C score in the present data. Also, no difference between age groups was found, and the factor structure was equal between both genders and age groups. However, since young, unmarried Saudi women were overrepresented these results should be interpreted with caution. Nevertheless, in age the present sample does reflect the composition of the Saudi Arabia population as it has a large young populations with a median age of 31.8 years and about 72% of the population is aged between 15 and 64 years (Worldometers, 2021; O'Neil, 2022).

Results regarding the psychometric properties of the BSQs adapted for use in Saudi Arabia are in line with other studies. The current study found a unidimensional factor structure and a high internal consistency, similar to previous western, Latin, and Iraqi-Arab studies (Pook, 2008; Welch et al., 2012; da Silva et al., 2014; Medya and Ishak, 2016), underlining the potential multi-cultural applicability of the BSQs. The BSQ8C can be used as a first screener before administration of the BSQ34 among those who scored above cut-off on the BSQ8C.

This study shows a variety of strengths. This study is the first to investigate body-shape dissatisfaction and the properties of the BSQs in a large Saudi community sample. Furthermore, the sample consisted of men and women, which is quite unique in Saudi society. As Saudi Arabia is socially a rather reclusive society (Melisse et al., 2022), being able to investigate such a large and diverse sample was a rare opportunity. Furthermore, as there was no effect of age, gender, and level of education, it may be concluded that both versions of the BSQ are widely applicable across Saudi Arabia to screen for or assess the severity of body-shape dissatisfaction. In addition, this study contributes to the assessment and knowledge regarding body-shape dissatisfaction in Saudi Arabia. The proposed cut-off values can be used to select Saudis for preventative programs aiming to avoid the development of eating disorder symptoms (Stice and Shaw, 2002).

There are certain limitations to this study. First, since the BSQ was only completed once, test–retest reliability could not be established. Secondly, this study did not include a clinical sample. Inclusion of a clinical sample suffering from body-shape dissatisfaction related to eating disorders would have helped to determine the discriminant validity. However, unfortunately, eating disorders are barely recognized in Saudi Arabia (Melisse et al., 2021), creating difficulties to study a clinical population. Thirdly, the EDE-SC was used to determine how well the Saudi-Arabic BSQs discriminated between Saudis with low and high levels of body-shape dissatisfaction. Use of this EDE subscale is not ideal since its factor structure is inconclusive (Byrne, 2010; Grilo et al., 2010; O'Brien et al., 2016; Burke et al., 2017). Examining the factor structure of the full Saudi-Arabic EDE would be superior. However, only 98 participants completed the EDE, therefore running a CFA in this sample would not yield valid results. Though use of the EDE-SC appeared most suitable since bias appeared to be reduced due to its investigator-based nature (Cooper and Fairburn, 1987), the shape concern subscale has the highest internal consistency (Burke et al., 2017) and there are no other standardized measures available to measure body-shape dissatisfaction in Saudi Arabia. Fourthly, since data collection ended, there have been several cultural changes in Saudi Arabia, such as transformations to empower women and modernize the relatively conservative Saudi society (Melisse et al., 2022). Nowadays, women no longer have to wear a traditional abaya which might influence body image and therefore rates of body-shape dissatisfaction (Dittmar, 2005). In addition, when conducting the interviews, Saudi Arabia applied a strict gender separation, and interviews were conducted by female assessors only. Therefore, potentially only progressive Saudi men participated in this study. It is further noteworthy, that, though no effect of gender or educational level was found, there was a gender and educational bias in the current sample. There was an overrepresentation of highly educated women compared to the general Saudi population which should be considered when interpreting the results and potentially impacts generalizability. Furthermore, as women tend to show higher body-shape dissatisfaction than men in general (Stice and Shaw, 2002), though not in the present sample, cut-off scores suggested in this study should be used with some caution.

Future studies should take the limitations of this study into account. Based on the current results, a logical next step for future research would be to examine test–retest reliability of the Saudi-Arabic BSQs (Polit and Yang, 2016). Furthermore, examination of body-shape dissatisfaction is more reliable among clinical samples, for example among Saudis with eating disorder symptoms. In the present study, the external criterion to evaluate the screening ability of the BSQs was a score above or below the community mean + 1SD on the EDE-SC. Examination of the factor structure of the full Saudi-Arabic EDE among a sufficient sample is recommended. An alternative approach would be to compare BSQs scores in a mixed community and clinical sample, e.g., Saudis seeking treatment for eating disorders and evaluate discriminative validity of the BSQs. However, body-shape dissatisfaction and eating disorders are rarely recognized and treated in Saudi clinics (Alkhadari et al., 2016; Melisse et al., 2020a). In addition, it would also be of interest to examine the psychometric properties of additional measures assessing body-shape dissatisfaction or body-shape concern, such as the Body Attitude Test (Probst et al., 1995), Body Uneasiness Test (Cuzzolaro et al., 2006), and the Body Appreciation Scale-2 (Tylka and Wood-Barcalow, 2015). In contrast to the other self-reports, the Body Appreciation Scale-2 measures positive body image (Tylka and Wood-Barcalow, 2015). Both, the Body Attitude Test (Probst et al., 1995) and Body Uneasiness Test (Cuzzolaro et al., 2006) have a stable multi-factor structure and look into different aspects of body-shape dissatisfaction; however, the Body Uneasiness Test involves significantly more items compared to the BSQ34 and the Body Attitude Test is only moderately correlated with the BSQ34 (Probst et al., 1997). Furthermore, it is recommended to validate the Saudi-Arabic BSQ34 among Saudis with excess weight, like the Body Uneasiness Test has been validated among patients with excess weight in other cultures (Marano et al., 2007). This is of relevance since almost half of the Saudi population suffers from excess weight and a high BMI is associated with more severe body-shape dissatisfaction in Saudi Arabia (Melisse et al., 2022). Moreover, it would also be of interest to investigate general psychopathology, as body-shape dissatisfaction was associated with increased levels of psychological symptoms (Murray et al., 2013; Gailledrat et al., 2016; Pritchard et al., 2021; Turk et al., 2021). Furthermore, body-shape dissatisfaction also predicted psychological symptoms in other cultures (Rodríguez-Cano et al., 2006; Rich and LeClere, 2011). Moreover, it would be of interest to investigate whether the recent transformations to modernize Saudi society, for example by releasing the obligation for women to wear an abaya, impacts body-shape dissatisfaction. For instance, body-shape dissatisfaction could be compared between women who still wear an abaya and women who have decided not to wear the traditional abaya anymore. Last, a more balanced community sample regarding gender, age, and educational level would increase confidence in the generalizability of the findings and normative values.

In conclusion, this study made a first attempt to evaluate the psychometric properties and provide preliminary normative data of Saudi-Arabic BSQs. Both, the BSQ34 and the BSQ8C displayed a unidimensional factor structure, high internal validity, and are, therefore, potentially valid assessment tools to measure body-shape dissatisfaction in Saudi Arabia. The estimated cut-off score for the BSQ34 was <114 and < 28 for the BSQ8C. Though no effect of gender, level of education and age was found on BSQs total score and the BSQs performed equally across gender and age, unmarried women were overrepresented in this study which potentially impacts generalizability of the Arabic BSQ. Therefore, the results should be interpreted with care when the BSQs are applied across Saudi Arabia.

## Data availability statement

The data and materials analyzed in this study are available upon reasonable request to the corresponding author.

## Ethics statement

The studies involving human participants were reviewed and approved by the ethical board of PNU, Riyadh, Saudi Arabia (17–0097). Study approval was given on May 7th, 2017. The patients/ participants provided their written informed consent to participate in this study.

## Author contributions

The manuscript has been written by BM in collaboration with EF and EB. BM was responsible for cultural adaptation of the measures and data collection. All authors contributed to the article and approved the submitted version.

## Conflict of interest

The authors declare that the research was conducted in the absence of any commercial or financial relationships that could be construed as a potential conflict of interest.

## Publisher’s note

All claims expressed in this article are solely those of the authors and do not necessarily represent those of their affiliated organizations, or those of the publisher, the editors and the reviewers. Any product that may be evaluated in this article, or claim that may be made by its manufacturer, is not guaranteed or endorsed by the publisher.
