# Supplementary material for: The Saudi-Arabic adaptation of the Body Shape Questionnaire (BSQ34): Psychometrics and norms of the full version and the short version (BSQ8C)
Source: Front Psychol. 2022 Dec 1;13:1046075. doi: 10.3389/fpsyg.2022.1046075 (PMC9754054; doi:10.3389/fpsyg.2022.1046075)
Supplement: Supplementary file 1 [file Data_Sheet_1.docx]

**Supplementary Table A**

Basic psychometrics of the Saudi- Arabic BSQ items and total scores

|  |  |  |  |  |  |  | % of responses | | | | | | |
| --- | --- | --- | --- | --- | --- | --- | --- | --- | --- | --- | --- | --- | --- |
| Items | *n* | *M* | SD | median | skewness | kurtosis | 1 | 2 | 3 | 4 | 5 | 6 | Missing |
|  |  |  |  |  |  |  |  |  |  |  |  |  |  |
| Item1 | 871 | 2.66 | 1.44 | 3 | 0.71 | -0.26 | 25 | 24 | 28 | 8 | 8 | 6 | 0 |
| Item2 | 871 | 3.28 | 1.80 | 3 | 0.21 | -1.29 | 23 | 15 | 19 | 15 | 9 | 19 | 0 |
| Item3 | 870 | 3.11 | 1.85 | 3 | 0.35 | -1.27 | 30 | 13 | 20 | 11 | 8 | 19 | 0 |
| Item4 | 871 | 3.86 | 1.85 | 4 | -0.16 | -1.41 | 15 | 13 | 18 | 13 | 8 | 33 | 0 |
| Item5 | 867 | 3.80 | 1.80 | 4 | -0.12 | -1.35 | 14 | 13 | 19 | 14 | 10 | 29 | 1 |
| Item6 | 870 | 3.31 | 1.79 | 3 | 0.22 | -1.26 | 22 | 15 | 22 | 12 | 10 | 20 | 0 |
| Item7 | 869 | 1.76 | 1.37 | 1 | **1.88** | **2.53** | 68 | 12 | 8 | 4 | 3 | 4 | 0 |
| Item8 | 867 | 1.54 | 1.18 | 1 | **2.42** | **5.29** | 77 | 7 | 8 | 3 | 2 | 3 | 1 |
| Item9 | 869 | 2.83 | 1.73 | 3 | 0.56 | -0.94 | 32 | 16 | 21 | 10 | 8 | 13 | 0 |
| Item10 | 868 | 2.63 | 1.78 | 2 | 0.73 | -0.84 | 42 | 14 | 16 | 9 | 7 | 12 | 1 |
| Item11 | 868 | 1.84 | 1.35 | 1 | **1.69** | **2.00** | 63 | 14 | 11 | 4 | 3 | 4 | 1 |
| Item12 | 869 | 2.75 | 1.67 | 3 | 0.61 | -0.84 | 33 | 17 | 21 | 10 | 9 | 10 | 0 |
| Item13 | 872 | 1.86 | 1.35 | 1 | **1.66** | **1.89** | 60 | 18 | 11 | 4 | 4 | 4 | 0 |
| Item14 | 869 | 2.79 | 1.73 | 2 | 0.64 | -0.88 | 32 | 19 | 19 | 9 | 8 | 13 | 0 |
| Item15 | 871 | 3.05 | 1.79 | 3 | 0.38 | -1.21 | 28 | 16 | 19 | 10 | 11 | 15 | 0 |
| Item16 | 867 | 2.39 | 1.78 | 1 | 0.97 | -0.47 | 52 | 10 | 15 | 6 | 5 | 12 | 1 |
| Item17 | 868 | 2.97 | 1.76 | 3 | 0.47 | -1.06 | 29 | 17 | 20 | 12 | 7 | 15 | 1 |
| Item18 | 869 | 1.74 | 1.33 | 1 | **1.87** | **2.52** | 68 | 12 | 9 | 4 | 4 | 4 | 0 |
| Item19 | 869 | 2.04 | 1.57 | 1 | **1.40** | **0.72** | 60 | 12 | 11 | 5 | 4 | 7 | 0 |
| Item20 | 870 | 2.50 | 1.65 | 2 | 0.90 | -0.40 | 39 | 20 | 17 | 7 | 7 | 10 | 0 |
| Item21 | 868 | 2.75 | 1.73 | 2 | 0.63 | -0.88 | 35 | 15 | 20 | 10 | 7 | 12 | 1 |
| Item22 | 870 | 2.91 | 1.83 | 3 | 0.49 | -1.16 | 34 | 14 | 19 | 9 | 9 | 16 | 0 |
| Item23 | 868 | 2.55 | 1.77 | 2 | 0.77 | -0.81 | 46 | 12 | 15 | 9 | 8 | 11 | 1 |
| Item24 | 866 | 2.87 | 1.81 | 3 | 0.53 | -1.11 | 34 | 15 | 18 | 9 | 9 | 15 | 1 |
| Item25 | 869 | 2.02 | 1.55 | 1 | 1.41 | 0.76 | 61 | 11 | 12 | 6 | 4 | 7 | 0 |
| Item26 | 867 | 1.37 | 1.04 | 1 | **3.06** | **8.68** | 85 | 5 | 3 | 3 | 2 | 2 | 1 |
| Item27 | 868 | 1.97 | 1.51 | 1 | **1.52** | **1.14** | 61 | 14 | 10 | 5 | 4 | 6 | 1 |
| Item28 | 861 | 3.05 | 1.82 | 3 | 0.40 | -1.22 | 29 | 16 | 19 | 11 | 9 | 17 | 1 |
| Item29 | 864 | 2.49 | 1.70 | 2 | 0.90 | -0.47 | 43 | 17 | 17 | 6 | 7 | 10 | 1 |
| Item30 | 864 | 2.92 | 1.74 | 3 | 0.49 | -1.03 | 30 | 15 | 23 | 8 | 10 | 13 | 1 |
| Item31 | 867 | 1.80 | 1.39 | 1 | **1.78** | **2.16** | 67 | 11 | 11 | 3 | 4 | 4 | 1 |
| Item32 | 868 | 1.37 | 0.98 | 1 | **2.88** | **7.74** | 85 | 4 | 5 | 3 | 2 | 1 | 1 |
| Item33 | 863 | 2.99 | 1.75 | 3 | 0.39 | -1.13 | 30 | 14 | 20 | 13 | 10 | 13 | 1 |
| Item34 | 862 | 3.85 | 1.83 | 4 | -0.21 | -1.36 | 16 | 11 | 18 | 12 | 13 | 30 | 1 |
| BSQ34 | 867 | 87.70 | 36.75 | 82 | 0.57 | -0.39 |  |  |  |  |  |  |  |
| BSQ8C | 846 | 21.34 | 9.69 | 19 | 0.59 | -0.42 |  |  |  |  |  |  |  |

† The indicators marked in bold type signify skewed and peaked items with many responses in the lowest response category.

**Supplementary Table B**

Cross walk from raw scores to *T*-scores and percentiles for the Saudi- Arabic BSQ34

| RS† | T‡ | PR§ |  | RS | T | PR |  | RS | T | PR |  | RS | T | PR |
| --- | --- | --- | --- | --- | --- | --- | --- | --- | --- | --- | --- | --- | --- | --- |
| 34 | 26.5 | -0.7 |  | 77 | 48.5 | 44.8 |  | 120 | 58.4 | 79.2 |  | 163 | 68.3 | 96.6 |
| 35 | 28.2 | 0.6 |  | 78 | 48.7 | 45.7 |  | 121 | 58.6 | 79.8 |  | 164 | 68.5 | 96.7 |
| 36 | 29.8 | 1.8 |  | 79 | 48.9 | 46.6 |  | 122 | 58.9 | 80.5 |  | 165 | 68.7 | 96.9 |
| 37 | 31.2 | 3.0 |  | 80 | 49.2 | 47.5 |  | 123 | 59.1 | 81.1 |  | 166 | 69.0 | 97.0 |
| 38 | 32.4 | 4.2 |  | 81 | 49.4 | 48.4 |  | 124 | 59.3 | 81.7 |  | 167 | 69.2 | 97.2 |
| 39 | 33.5 | 5.4 |  | 82 | 49.6 | 49.3 |  | 125 | 59.5 | 82.3 |  | 168 | 69.4 | 97.3 |
| 40 | 34.5 | 6.6 |  | 83 | 49.9 | 50.2 |  | 126 | 59.8 | 82.8 |  | 169 | 69.7 | 97.4 |
| 41 | 35.4 | 7.8 |  | 84 | 50.1 | 51.1 |  | 127 | 60.0 | 83.4 |  | 170 | 69.9 | 97.5 |
| 42 | 36.2 | 9.0 |  | 85 | 50.3 | 52.0 |  | 128 | 60.2 | 84.0 |  | 171 | 70.1 | 97.6 |
| 43 | 37.0 | 10.1 |  | 86 | 50.6 | 52.9 |  | 129 | 60.5 | 84.5 |  | 172 | 70.4 | 97.7 |
| 44 | 37.7 | 11.2 |  | 87 | 50.8 | 53.8 |  | 130 | 60.7 | 85.1 |  | 173 | 70.6 | 97.8 |
| 45 | 38.3 | 12.4 |  | 88 | 51.0 | 54.7 |  | 131 | 60.9 | 85.6 |  | 174 | 70.8 | 97.9 |
| 46 | 38.9 | 13.5 |  | 89 | 51.3 | 55.5 |  | 132 | 61.2 | 86.1 |  | 175 | 71.0 | 98.0 |
| 47 | 39.4 | 14.6 |  | 90 | 51.5 | 56.4 |  | 133 | 61.4 | 86.6 |  | 176 | 71.3 | 98.0 |
| 48 | 39.9 | 15.7 |  | 91 | 51.7 | 57.3 |  | 134 | 61.6 | 87.1 |  | 177 | 71.5 | 98.1 |
| 49 | 40.4 | 16.8 |  | 92 | 52.0 | 58.1 |  | 135 | 61.8 | 87.6 |  | 178 | 71.7 | 98.1 |
| 50 | 40.8 | 17.9 |  | 93 | 52.2 | 59.0 |  | 136 | 62.1 | 88.1 |  | 179 | 72.0 | 98.2 |
| 51 | 41.2 | 19.0 |  | 94 | 52.4 | 59.8 |  | 137 | 62.3 | 88.5 |  | 180 | 72.2 | 98.2 |
| 52 | 41.6 | 20.0 |  | 95 | 52.6 | 60.6 |  | 138 | 62.5 | 89.0 |  | 181 | 72.4 | 98.3 |
| 53 | 42.0 | 21.1 |  | 96 | 52.9 | 61.5 |  | 139 | 62.8 | 89.4 |  | 182 | 72.7 | 98.3 |
| 54 | 42.4 | 22.1 |  | 97 | 53.1 | 62.3 |  | 140 | 63.0 | 89.9 |  | 183 | 72.9 | 98.4 |
| 55 | 42.7 | 23.2 |  | 98 | 53.3 | 63.1 |  | 141 | 63.2 | 90.3 |  | 184 | 73.1 | 98.4 |
| 56 | 43.0 | 24.2 |  | 99 | 53.6 | 63.9 |  | 142 | 63.5 | 90.7 |  | 185 | 73.3 | 98.4 |
| 57 | 43.3 | 25.3 |  | 100 | 53.8 | 64.7 |  | 143 | 63.7 | 91.1 |  | 186 | 73.6 | 98.4 |
| 58 | 43.6 | 26.3 |  | 101 | 54.0 | 65.5 |  | 144 | 63.9 | 91.4 |  | 187 | 73.8 | 98.5 |
| 59 | 43.9 | 27.3 |  | 102 | 54.3 | 66.3 |  | 145 | 64.1 | 91.8 |  | 188 | 74.0 | 98.5 |
| 60 | 44.2 | 28.3 |  | 103 | 54.5 | 67.1 |  | 146 | 64.4 | 92.2 |  | 189 | 74.3 | 98.5 |
| 61 | 44.5 | 29.3 |  | 104 | 54.7 | 67.9 |  | 147 | 64.6 | 92.5 |  | 190 | 74.5 | 98.6 |
| 62 | 44.8 | 30.3 |  | 105 | 54.9 | 68.6 |  | 148 | 64.8 | 92.9 |  | 191 | 74.7 | 98.6 |
| 63 | 45.0 | 31.3 |  | 106 | 55.2 | 69.4 |  | 149 | 65.1 | 93.2 |  | 192 | 75.0 | 98.6 |
| 64 | 45.3 | 32.3 |  | 107 | 55.4 | 70.2 |  | 150 | 65.3 | 93.5 |  | 193 | 75.2 | 98.7 |
| 65 | 45.5 | 33.3 |  | 108 | 55.6 | 70.9 |  | 151 | 65.5 | 93.8 |  | 194 | 75.4 | 98.7 |
| 66 | 45.8 | 34.3 |  | 109 | 55.9 | 71.6 |  | 152 | 65.8 | 94.1 |  | 195 | 75.6 | 98.7 |
| 67 | 46.1 | 35.3 |  | 110 | 56.1 | 72.4 |  | 153 | 66.0 | 94.4 |  | 196 | 75.9 | 98.8 |
| 68 | 46.3 | 36.2 |  | 111 | 56.3 | 73.1 |  | 154 | 66.2 | 94.6 |  | 197 | 76.1 | 98.9 |
| 69 | 46.6 | 37.2 |  | 112 | 56.6 | 73.8 |  | 155 | 66.4 | 94.9 |  | 198 | 76.3 | 98.9 |
| 70 | 46.8 | 38.2 |  | 113 | 56.8 | 74.5 |  | 156 | 66.7 | 95.1 |  | 199 | 76.6 | 99.0 |
| 71 | 47.0 | 39.1 |  | 114 | 57.0 | 75.2 |  | 157 | 66.9 | 95.4 |  | 200 | 76.8 | 99.1 |
| 72 | 47.3 | 40.1 |  | 115 | 57.2 | 75.9 |  | 158 | 67.1 | 95.6 |  | 201 | 77.0 | 99.1 |
| 73 | 47.5 | 41.0 |  | 116 | 57.5 | 76.6 |  | 159 | 67.4 | 95.8 |  | 202 | 77.3 | 99.2 |
| 74 | 47.8 | 42.0 |  | 117 | 57.7 | 77.2 |  | 160 | 67.6 | 96.0 |  | 203 | 77.5 | 99.4 |
| 75 | 48.0 | 42.9 |  | 118 | 57.9 | 77.9 |  | 161 | 67.8 | 96.2 |  | 204 | 77.7 | 99.5 |
| 76 | 48.2 | 43.8 |  | 119 | 58.2 | 78.6 |  | 162 | 68.1 | 96.4 |  |  |  |  |

† Raw scores;

‡ Calculated *T*-scores based on IRT (*T* =30.8-EXP(-0.132*RS-52.9))+0.23*RS, an exponential function);

§ Calculated Percentile Rank Scores (*T*=-56,7+2,218*RS-2,314e-02*RS^2+2,238e-04*RS^3-1,135e-06*RS^4+2,073e-09*RS^5, a polynomial function).

**Supplementary Table C**

Cross walk from raw scores to *T*-scores and Percentile ranks scores

for the Saudi- Arabic BSQ8C.

| RS† | *T*‡ | PR§ |  | RS† | *T*‡ | PR§ |  | RS† | *T*‡ | PR§ |
| --- | --- | --- | --- | --- | --- | --- | --- | --- | --- | --- |
| 8 | 34.2 | 3.1 |  | 22 | 52.1 | 57.4 |  | 36 | 61.9 | 90.9 |
| 9 | 36.1 | 7.3 |  | 23 | 52.9 | 60.6 |  | 37 | 62.7 | 92.3 |
| 10 | 37.8 | 11.5 |  | 24 | 53.6 | 63.7 |  | 38 | 63.5 | 93.5 |
| 11 | 39.5 | 15.7 |  | 25 | 54.4 | 66.7 |  | 39 | 64.4 | 94.6 |
| 12 | 41.0 | 19.8 |  | 26 | 55.1 | 69.6 |  | 40 | 65.3 | 95.6 |
| 13 | 42.5 | 23.9 |  | 27 | 55.7 | 72.3 |  | 41 | 66.3 | 96.5 |
| 14 | 43.8 | 27.9 |  | 28 | 56.4 | 75.0 |  | 42 | 67.4 | 97.2 |
| 15 | 45.1 | 31.9 |  | 29 | 57.1 | 77.4 |  | 43 | 68.5 | 97.8 |
| 16 | 46.3 | 35.8 |  | 30 | 57.7 | 79.8 |  | 44 | 69.7 | 98.3 |
| 17 | 47.4 | 39.6 |  | 31 | 58.4 | 82.0 |  | 45 | 70.9 | 98.6 |
| 18 | 48.5 | 43.4 |  | 32 | 59.1 | 84.0 |  | 46 | 72.3 | 98.9 |
| 19 | 49.5 | 47.0 |  | 33 | 59.7 | 86.0 |  | 47 | 73.7 | 99.0 |
| 20 | 50.4 | 50.6 |  | 34 | 60.4 | 87.8 |  | 48 | 75.3 | 99.1 |
| 21 | 51.3 | 54.0 |  | 35 | 61.2 | 89.4 |  |  |  |  |

† Raw scores;

‡ Calculated T-scores based on IRT (*T*=14.6+3.040*RS+8.067e-02*RS^2+9.096e-04*RS^3, a hyperbolic function);

§ Calculated Percentile Rank Scores (*T*=-29,8+3,879*RS+4,679e-02*RS^2-2,342e-03*RS^3+1,769e-05*RS^4, a polynomial function).
